# Hidden α-helical propensity segments within disordered regions of the transcriptional activator CHOP

**DOI:** 10.1371/journal.pone.0189171

**Published:** 2017-12-06

**Authors:** Ángeles Canales, Marcel Rösinger, Javier Sastre, Isabella C. Felli, Jesús Jiménez-Barbero, Guillermo Giménez-Gallego, Carlos Fernández-Tornero

**Affiliations:** 1 Departamento de Química Orgánica I, Universidad Complutense de Madrid, Madrid, Spain; 2 Centro de Investigaciones Biológicas, CSIC, Madrid, Spain; 3 Magnetic Resonance Center & Department of Chemistry, University of Florence, Sesto Fiorentino, Italy; Russian Academy of Medical Sciences, RUSSIAN FEDERATION

## Abstract

C/EBP-homologous protein (CHOP) is a key determinant of the apoptotic response to endoplasmic reticulum stress or DNA damage. As a member of the C/EBP family, CHOP contains a low complexity N-terminal region involved in transcriptional activation, followed by a bZIP that binds DNA after dimerization. However, in contrast to other C/EBPs, CHOP directs binding to non-canonical C/EBP sites due to unique substitutions in its DNA-binding domain. Herein, we show that the N-terminal region of CHOP is intrinsically unstructured but contains two segments presenting α-helical propensity. One of these segments is conserved in other C/EBPs and mediates essential roles of CHOP, including regulation through phosphorylation. The second segment is placed within a proteolytic-resistant portion of the protein and exhibits reduced flexibility. Moreover, the DNA-binding region of CHOP also contains a segment with α-helical character towards its most N-terminal part. Our results suggest that structure-prone segments scattered within disordered regions may be critical for macromolecular recognition during CHOP-mediated transcriptional activation.

## Introduction

C/EBP-homologous protein (CHOP), also known as C/EBPζ, DNA damage-inducible transcript 3 protein (DDIT3) or Growth arrest and DNA damage-inducible protein (GADD153), is a basic leucine zipper (bZIP) belonging to the CCAAT/enhancer-binding protein (C/EBP) family of transcriptional activators. CHOP is ubiquitously expressed at low basal levels but, after endoplasmic reticulum (ER) stress or DNA damage, its expression significantly increases to induce growth arrest and apoptosis [1]. As ER stress-mediated apoptosis is associated with diseases like diabetes, ischemia and neurodegenerative disorders, CHOP plays a role in many pathogenic processes [2]. Moreover, a chromosomal translocation that fuses CHOP to the N-terminal region of the RNA-binding protein FUS occurs at the origin of malignant liposarcoma [3, 4].

**Fig 1:**
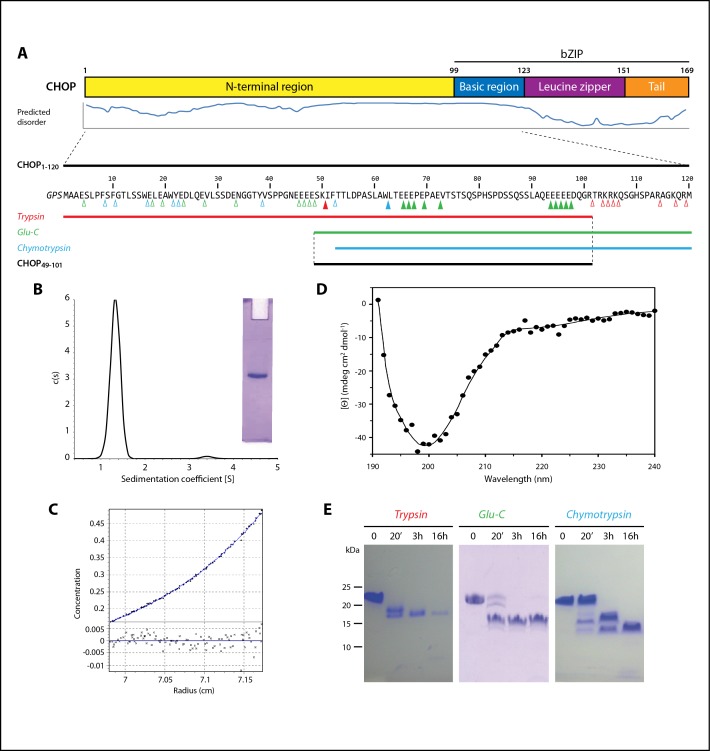
Biochemical characterization of the intrinsically disordered regions of CHOP. (A) Bar diagram of CHOP domains with level of disorder according to Disopred [15]. Below, sequence of our CHOP_1-120_ construct with red, green and blue triangles marking possible cleavage sites for trypsin, Glu-C and chymotrypsin, respectively. Empty and filled triangles indicate protease-accessible and protease-protected positions, respectively. Coloured bars represent the fragments resulting from proteolysis experiments, while the bottom black bar shows the fragment obtained from the combined action of trypsin and Glu-C. (B) Sedimentation velocity and native PAGE (inset), of CHOP_1-120_, showing that the sample is monodisperse. (C) Sedimentation equilibrium of the same sample. (D) Circular dichroism spectrum of CHOP_1-120_. Experimental measurements of the sample after subtraction of the buffer signal are shown as dots, with an smoothen curve as delivered by Dichroweb (http://dichroweb.cryst.bbk.ac.uk/). (E) Time-course proteolytic digestion of CHOP_1-120_ as seen by Tris-tricine PAGE.

**Fig 2:**
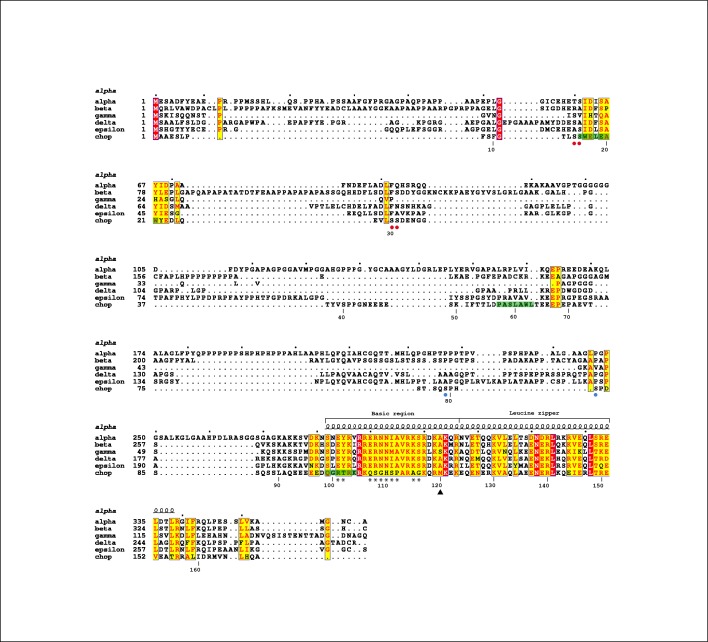
Alignment of the human C/EBP family. Fully- and partially-conserved residues are in red and yellow, respectively. The secondary structure of the C/EBPα bZIP domain is shown above the sequences, while the numbering below corresponds to CHOP. The segments with α-helical propensity as identified by NMR (see below) are in green. Red and blue circles below the sequence indicate inactivating and activating phosphorylations in CHOP [7,8], while asterisks highlight unique changes in its DNA-binding domain. Figure prepared with ESPript (http://espript.ibcp.fr).

CHOP is composed by a non-conserved N-terminal region, likely containing activation domains (AD) for interaction with other transcriptional regulators, followed by a conserved bZIP that comprises a basic DNA-binding region and a leucine zipper involved in dimerization, plus a C-terminal tail (Fig 1A). Like for other C/EBPs, the N-terminal region of CHOP exhibits low complexity and is, thus, considered to be intrinsically unstructured. This region has been reported to interact with the coactivator protein p300 and the transcriptional repressor TRB3 [5, 6]. Moreover, the N-terminal region of CHOP was shown to regulate the transcriptional activity of CHOP through phosphorylation [7, 8]. In contrast to the N-terminal region, the bZIP domain of CHOP is well conserved with other C/EBPs (Fig 2). Therefore, in spite of a few changes at positions that are critical for DNA-binding [9], the bZIP domain of CHOP is expected to adopt an α-helical fold in the presence of DNA, as shown for the homodimerized bZIP domains of C/EBPα and C/EBPβ [10, 11]. However, in the absence of its DNA target, the basic region of bZIP proteins is significantly unfolded in solution [12], as also shown for C/EBPα [13]. In agreement, full-length CHOP in the absence of DNA was shown to behave as an intrinsically disordered protein (IDP) [14].

IDPs are characterized by the lack of well-defined tertiary structure, which correlates with conformational heterogeneity [16]. These proteins and regions are generally involved in the interaction with other macromolecules to regulate critical cellular processes [17, 18]. Binding of IDPs to their targets is often associated with folding [19], following the induced-fit paradigm. The existence in IDPs of segments with secondary structure propensity has been proposed to favor this kind of interactions [20]. A few pre-formed secondary structure elements would nucleate binding while the remaining flexible parts would follow.

In spite of its critical role in the control of mammalian gene expression, structural studies on CHOP are scarce. Here, we have combined biochemical and biophysical methods, including advanced nuclear magnetic resonance experiments, to obtain novel structural information on the N-terminal and basic regions of CHOP, both associated with a disordered state in the absence of binding macromolecules. These studies allowed us to identify segments with α-helical propensity within both regions. Based on our data, we propose that such segments represent nucleation points to guide the interaction of CHOP with its binding partners.

## Materials and methods

### Protein expression and purification

The DNA segment coding for the predicted unstructured region of human CHOP, residues 1 to 120, was introduced in the pRAT-4 vector [21] following standard methods. A tandem affinity tag constituted by a histidine tag followed by a choline-binding domain [22] was appended at the N-terminus of the construct, using the 3C Protease cleavage sequence as linker. The resulting plasmid was transformed into *E*. *coli* BL21(DE3), kind gift of F. W. Studier. A 200 ml culture was grown at 37°C in LB supplemented with 0.1 mg/ml ampicillin, pelleted at 4°C and 5,000 g for 4 minutes, washed twice in M9 minimal medium, and used to inoculate 5 L of M9 with 0.1 mg/ml ampicillin. When the culture reached an OD_600_ of 0.8, protein expression was induced with 0.5 mM IPTG. After overnight expression at 37°C, the culture was centrifuged at 4°C and 7,000 g for 20 minutes, and stored at -80°C. For expression in labeled medium, the same procedure was followed, substituting NH_4_Cl and glucose with their corresponding ^15^N and ^13^C isotopes (Cortecnet).

Cells were resuspended in HT-buffer (50 mM Tris-HCl pH 7.6, 0.5 M NaCl, 12 mM imidazole) supplemented with 4 mM MgCl_2_, 1 mg/ml lysozyme, DNase I (Roche) and cOmplete protease inhibitors (Roche). Cells were lysed with a French Press, centrifuged at 4°C and 55,000 g for 60 minutes. The supernatant was loaded on a HisTrap column (GE Healthcare) equilibrated in HT-buffer and gradient-eluted in the same buffer containing 500 mM imidazole. Selected fractions were dialyzed against MQ-buffer (20 mM Tris-HCl pH 7.6, 100 mM NaCl) and incubated with non-commercial 3C protease at a 1:100 ratio and 4°C. The sample was then loaded on a mono-Q column (GE Healthcare) and gradient-eluted in the same buffer containing 1 M NaCl. Selected fractions were injected on a Superose-12 column (GE Healthcare) equilibrated in SE-buffer (10 mM Tris-HCl pH 7.6, 50 mM NaCl). The purified protein was concentrated to 14 mg/ml and stored -80°C for further use. For circular dichroism and NMR experiments, the buffer was exchanged to CD-buffer (10 mM phosphate pH 7.1, 50 mM NaCl) using Amicon centrifugal filters (Millipore) with 3kDa cutoff.

### Analytical ultracentrifugation

Experiments were conducted at 20°C using an AnTi50 eight-hole rotor, epon-charcoal standard double sector centerpieces (12 mm optical path) and an Optima XL-I analytical ultracentrifuge (Beckman-Coulter). The protein was used at 1.08 mg/ml and absorbance was measured at 280 nm. Sedimentation velocity experiments were performed at 48 krpm using 400 μL samples. Differential sedimentation coefficient distributions, c(s), were calculated by least-squares boundary modeling of sedimentation velocity data using the program Sedfit [23]. From this analysis, the experimental sedimentation coefficients were corrected for solvent composition and temperature with program Sednterp [24] to obtain the corresponding standard s-values. Short-column (85 μL) sedimentation equilibrium runs were carried out at multiple speeds, 20 and 24 krpm. After equilibrium scans, a high-speed centrifugation run at 48 krpm was done to estimate the corresponding baseline offsets. Weight-average buoyant molecular weights were determined by fitting a single species model to the experimental data using the Hetero-Analysis [25]. The molecular weight of the protein was determined from the experimental buoyant values using 0.707 ml/g as their partial specific volumes, calculated from the amino acid composition using Sednterp.

### Circular dichroism

All data were acquired on a Jasco J-815 Spectropolarimeter at 25°C using the software provided by Jasco. Measurements were performed for 20 μM of protein in CD-buffer, using cells with 0.1 mm optical path. Ten scans at 0.2 nm steps were accumulated, averaged and baseline-corrected for buffer to obtain the final plot.

### Limited proteolysis

Experiments were performed with sequencing-grade proteases, including trypsin (Roche), Glu-C (Promega) and chymotrypsin (Boehringer-Mannheim). For peptide identification, digestions were performed at 4°C, with a protein concentration of 0.5 mg/ml and a protease-to-target ratio of 1:100, in PD-buffer (10 mM Tris-HCl pH 7.6, 50 mM NaCl, 10 mM MgCl_2_). For large-scale preparation of the proteolytically-resistant fragment, CHOP_1-120_ was first incubated with Glu-C at 25°C for 20 minutes, then with trypsin at 4°C for 3 hours. Digested peptides were bound to a ZORBAX 300 Extend-C18 column (Agilent) equilibrated with 0.01% TFA in water, then gradient-eluted with 0.009% TFA in 60% acetonitrile/30% isopropanol. Samples were lyophilized and stored at -80°C for further use.

### Nuclear magnetic resonance

^13^C direct detected experiments were acquired with a 1 mM sample of the protein in CD-buffer containing 90% H_2_O and 10% D_2_O. 2D CON, 3D CBCACON and 3D CBCANCO spectra where measured in a 900 MHz Bruker spectrometer. 3D CBCACON was acquired with 8 scans and spectral widths of 49, 36 and 60 ppm for ^13^C, ^15^N and ^13^C, respectively. 3D CBCANCO was acquired with 16 scans and the same spectral widths as CBCACON. Standard proton detected 3D HNCO, 3D HN(CA)CO, 3D HNCACB, 3D HN(CO)CACB and 3D HN(CA)NNH spectra were acquired in a 700 MHz Bruker spectrometer to complete the assignment. HNCO was collected with 2 scans and HN(CA)CO was collected with 4 scans and spectral widths of 15.9, 26 and 10 ppm for ^1^H, ^15^N and ^13^C, respectively. 3D HNCACB was acquired with 8 scans and 3D HN(CO)CACB was acquired with 4 scans and spectral widths of 15.9, 26 and 60 ppm for ^1^H, ^15^N and ^13^C, respectively. 3D HN(CA)NNH spectrum was acquired with 32 scans and spectral widths of 15.9, 26 and 26 ppm for ^1^H, ^15^N and ^15^N, respectively.

^15^N T_1_, T_2_, and ^1^H-^15^N nuclear Overhausser effect (NOE) experiments were acquired with a 1 mM sample of a proteolysis-derived fragment in CD-buffer containing 90% H_2_O and 10% D_2_O, using a Bruker Advance 700 MHz spectrometer. Experimentally, 10 data points were acquired interleaved with relaxation delays in the range 20 ms-1.5 s (20, 100, 200, 350, 500, 600, 800, 1000, 1200 and 1500 ms) for T_1_ or 31.36 ms-0.47 s (31.36, 62.72, 94.08, 125.44, 156.80, 219.52, 283, 344.96, 407.68 and 470.40 ms) for T_2_, using a delay between experiments of 3s. T_2_ was measured using a 0.9 ms delay between refocusing pulses in the CPMG train. Heteronuclear NOE was measured in an interleaved manner using a ^1^H saturation time of 3s and a relaxation delay of 3s. The spectral widths are 15.9 and 22 ppm for ^1^H and ^15^N dimensions, respectively. All of the experiments were acquired at a temperature of 298 K. T_1_ and T_2_ values were obtained by fitting the decay of intensity of each peak to an exponential function as implemented in the software Dynamic Center (Bruker). CLEANEX experiments were acquired on a Bruker 700 MHz spectrometer with 16 scans and spectral widths of 15.9 and 22 ppm for ^1^H and ^15^N, respectively. Four filter delays 6, 12, 18 and 24 ms were used.

## Results

### The disordered region of CHOP contains a proteolytically-resistant portion

We first aimed to characterize the N-terminal region of CHOP biochemically. This region exhibits a high content of glutamate (20%), serine (17%) and proline (8%), which is typical of intrinsically disordered proteins [26]. Moreover, disorder prediction algorithms suggested that this region and also the basic region of CHOP are likely unstructured in solution (Fig 1A). We expressed and purified a construct that includes both the N-terminal and basic regions of CHOP, hereafter named CHOP_1-120_ (Fig 1A). As it lacks the leucine zipper, this construct is expected to behave as a monomer in solution. Both native gel electrophoresis and sedimentation velocity experiments showed that the purified construct is monodisperse (Fig 1B). Moreover, sedimentation equilibrium experiments indicated a molecular mass of 15.8 kDa (Fig 1C), in agreement with the expected value for a monomer of the purified protein (13.5 kDa). The small discrepancy in mass is likely due to the fact that our construct is indeed intrinsically disordered. This is confirmed by circular dichroism studies showing that CHOP_1-120_ presents the typical spectrum of unstructured proteins (Fig 1D). These proteins exhibit a shallow negative signal from 240 to 215 nm and a strong negative contribution thereafter with a minimum just below 200 nm [27], as is the case for CHOP_1-120_. Finally, our construct presents delayed migration on SDS-PAGE by a factor of about 1.6 (Fig 1E, t = 0), which is within the typical range of other IDPs [18].

We employed limited proteolysis to explore if the N-terminal domain of CHOP contains a certain degree of higher-order structure. Time course digestion with three different endopeptidases yielded stable fragments in all cases (Fig 1E). Electrophoretic mobility of CHOP_1-120_ and derived peptides is slower than expected, likely due to their overall acidic character (20% of acidic residues in CHOP_1-120_). We then purified the proteolytic fragments by reversed-phase chromatography and combined mass spectrometry with N-terminal sequencing to derive their sequence (Fig 1A). Trypsin yielded the largest fragment, which comprises residues 1 to 101 and contains one target bond after residue 50 that is not cleaved by the protease. As expected from its high content in basic residues, the DNA-binding domain of CHOP is readily digested by trypsin. Chymotrypsin produced the shortest fragment, spanning residues 53 to 120, which also contains one target bond after residue 61 that is not cleaved by the protease. The fragment derived from endopeptidase Glu-C digestion includes residues 49 to 120 and contains 10 target bonds that are not cleaved by the protease. These experiments suggest that partial ordering may occur in the C-terminal half of the CHOP N-terminal region. Therefore, we combined trypsin and Glu-C to produce the C-terminal half of CHOP N-terminal region, comprising residues 49 to 101, hereafter named CHOP_49-101_.

### The disordered region of CHOP contains three segments with α-helical propensity

**Fig 3:**
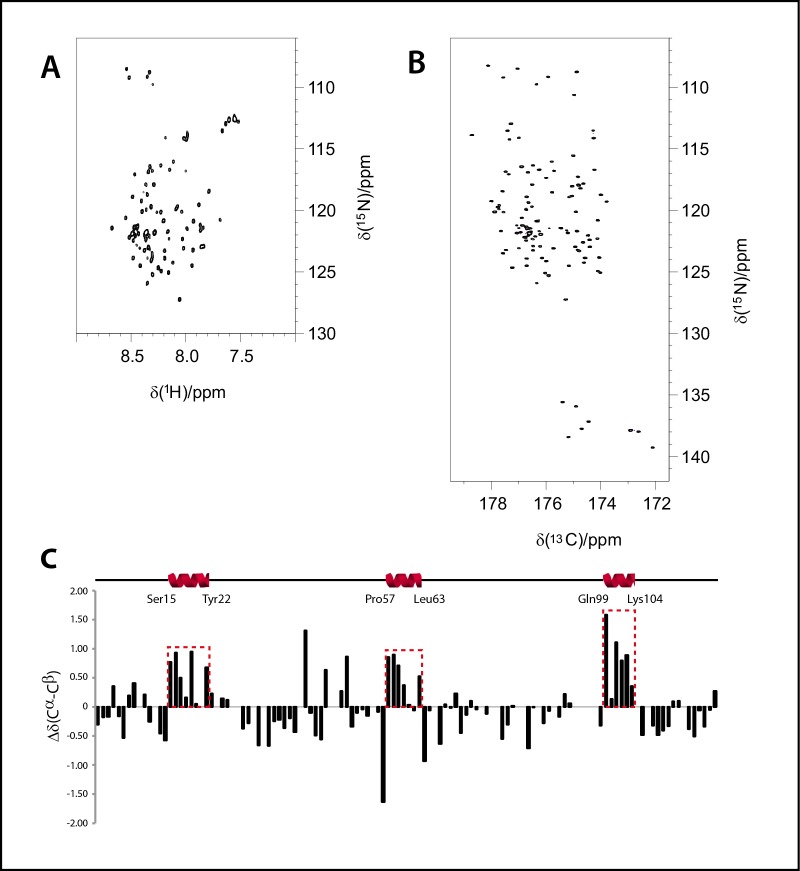
α-helical segments in the intrinsically disordered regions of CHOP. (A) ^1^H-^15^N HSQC spectrum of our CHOP_1-120_ construct. (B) CON spectrum of the same sample. (C) Plots of the observed differences in experimental chemical shifts (Cα–Cβ) with respect to those predicted for a random coil. The data have been corrected by considering the contribution of the corresponding primary sequence, using random coil chemical shifts for intrinsically disordered proteins (http://www1.bio.ku.dk/english/research/bms/research/sbinlab/groups/mak/randomcoil/script/). A schematic representation of the segments with α-helical propensity is shown above.

We then sought to unravel the possible structural features behind proteolytic protection by analysing the N-terminal and basic regions of CHOP. Due to their overall intrinsically disordered character, we employed nuclear magnetic resonance (NMR). Thus, we initially labelled our CHOP_1-120_ construct with ^15^N. The ^1^H-^15^N HSQC spectrum displayed all the observed backbone NH signals within a very narrow area in the ^1^H dimension (8.6–7.4 ppm). This fact strongly suggests that this protein is predominantly disordered in solution (Fig 3A). Disordered proteins represent challenging systems for conventional NMR methods due to intense signal overlap and fast HN-water exchange processes. However, in the last years, the introduction of ^13^C-direct detection experiments has allowed to circumvent these problems, given the larger heteronuclear chemical shift dispersion and the use of non-exchangeable nuclei for the coherence transfer events [28].

Therefore, we labelled our CHOP_1-120_ construct with ^15^N and ^13^C and acquired a CON spectrum, which correlates the nitrogen resonance of a certain residue with the carbonyl of the previous one. The intrinsically higher dispersion of the carbonyl chemical shifts provides a spectacular improvement in signal dispersion (Fig 3B). In addition, proline residues that are usually abundant in disordered proteins can also be easily detected. Following this ^13^C-detected approach, it was possible to acquire a set of heteronuclear 3D-NMR experiments for sequential assignment. The combination of CBCANCO and CBCACON spectra allowed us to derive most of the Cα and Cβ assignments. Standard 3D HNCO, 3D HN(CA)CO, 3D HNCACB, 3D HN(CO)CACB and 3D HN(CA)NNH were also acquired to complete the assignment process. Thanks to the information provided by these spectra, 88% of the backbone resonances were assigned (S1 Table).

In a further step, the identified Cα and Cβ chemical shifts were used to predict the secondary structure propensities of the protein by comparing the experimental chemical shifts with those expected for a random coil peptide (Fig 3C). Interestingly, despite the fact that CHOP_1-120_ is highly disordered in solution, we were able to identify three protein segments showing propensity to form short α-helices. In particular, these segments comprise residues 15–22 (-SWELEAWY-), 57–62 (-PASLAWL-) and 99–104 (-QGRTRK-). The latter corresponds to the first part of the DNA-binding domain, which we expected to present α-helical character due to partial homology with crystallized C/EBPs when bound to DNA [10, 11]. The other two segments, comprising residues 15–22 and 57–62, are located in the CHOP N-terminal region. Because this region is involved in binding other transcriptional regulators [5,6], the identified segments may represent hotspots for protein-protein interaction.

To explore this possibility, we applied bioinformatic tools developed to predict, within IDPs, molecular recognition features (MoRFs) that may undergo disorder-to-order transitions upon binding. Both MoRFpred [29] and ANCHOR [30] detect three equivalent MoRFs, while a fourth feature is only identified in ANCHOR with lower probability (S1 Fig). Interestingly, two MoRFs predicted by both programs (residues 10–24 and 52–66 in MoRFpred) include to the two segments with α-helical propensity in the N-terminal region of CHOP. The third MoRF predicted by both programs (residues 112–120 in MoRFpred) locates in the DNA binding domain and is adjacent to the third segment with α-helical propensity detected by NMR (residues 99–104), which localizes in the same region of the protein.

### α-helical propensity correlates with reduced flexibility

The segment with α-helical propensity comprising residues 57–62 is contained within CHOP_49-101_, the fragment resulting from proteolytic cleavage of CHOP_1-120_. While the α-helical character of the three segments described above is firmly established, the availability of this proteolytic fragment provides the opportunity of getting further insights into these regions using it as paradigm. To that purpose, we performed NMR relaxation experiments on the ^15^N-labelled CHOP_49-101_. Longitudinal (T_1_) and transverse (T_2_) relaxation times for the ^15^N amide backbone residues, as well as ^1^H-^15^N heteronuclear NOEs, were used to identify individual residues displaying fast motional properties. For flexible proteins, the heteronuclear NOE is the most sensitive reporter of fast local motions and provides direct information on the regions of the protein with high flexibility. Heteronuclear NOE values span a large range (from -4 to +1) in proteins with disorder regions. The obtained values can be combined with the analysis of the transverse relaxation rates that, in addition to the fluctuations depending on the local correlation time, are also sensitive to exchange processes.

**Fig 4:**
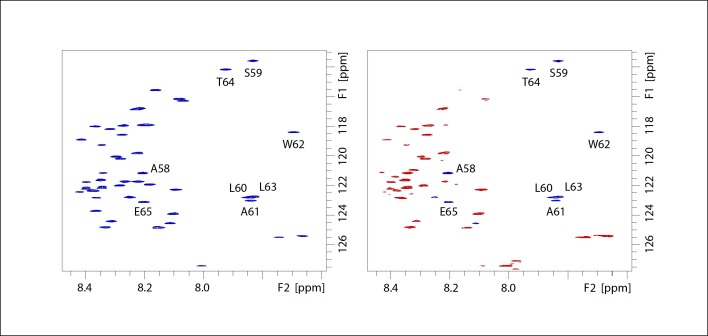
Intrinsic flexibility of the CHOP proteolytic fragment. Spectra acquired for the measurement of heteronuclear ^1^H-^15^N NOE. Reference (left) and NOE-containing (right) spectra with negative and positive cross-peaks in red and blue, respectively.

**Fig 5:**
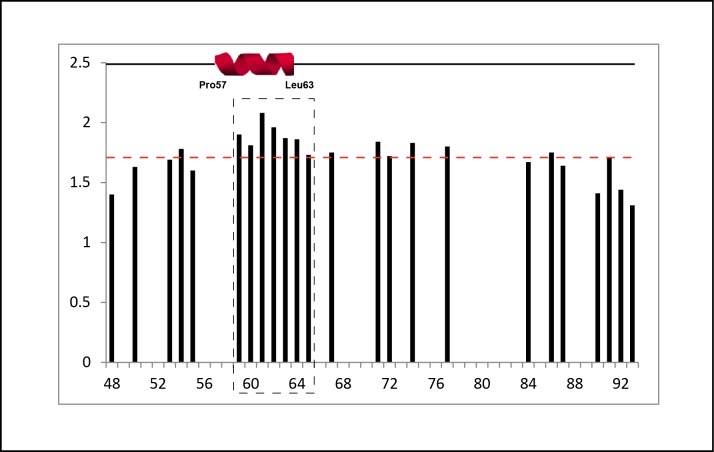
^15^N NMR relaxation data. T_1_/T_2_ ratio along the peptide sequence of CHOP_49-101_ showing that residues 59–65 display a slightly higher value than the average. A schematic representation of the segment with α-helical propensity is shown above.

Negative heteronuclear NOE values were obtained for most of the residues, in agreement with high flexibility of CHOP_49-101_ (Fig 4). However, there was also a well-defined segment with positive NOE values. This sequence comprises residues 59–65 (-SLAWLTE-), in agreement with the segment shown to display α-helical propensity in the chemical shift analysis (residues 57–62). In addition, the measured T_1_/T_2_ ratio was slightly larger for the segment comprising residues 59–65 than for the rest of the proteolytic peptide (Fig 5). These data indicate that the motional freedom of this segment is more restricted than the rest of the peptide.

**Fig 6:**
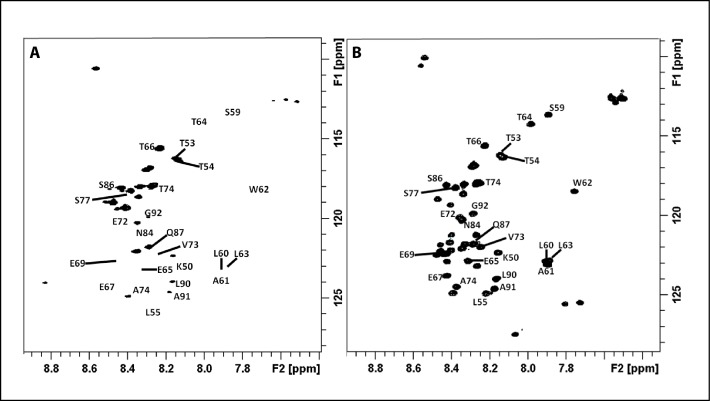
Solvent exposure data. (A) CLEANEX filtered experiment of CHOP_49-101_ (mixing time of 24 ms) where residues 59–65 are very weak, thus confirming that this segment is less exposed to the solvent. (B) HSQC of CHOP_49-101_ for comparison.

Finally, CLEANEX exchange experiments were also acquired. These NMR experiments allow detecting protein regions that are more exposed to the solvent and therefore more prone to exchange processes. At increasing mixing times, the cross peak intensities gradually increase, due to the HN exchange with the solvent. Regions located in secondary structure elements are less prone to solvent exchange and can be identified by their weak cross peak intensities in these experiments, as is the case for all HN signals belonging to the segment comprising residues 59–65, strongly suggesting that these HN are more protected to water exchange than the other backbone HN analogues (Fig 6). Overall, our results show that a well-defined segment in the C-terminal half of the CHOP N-terminal region (residues 49–101) displays a clear α-helical propensity, also characterized by the observed restricted motion in solution.

## Discussion

A partially unstructured N-terminal region of CHOP matches with the general idea that activation domains of transcription factors are highly dynamic and only adopt a tertiary structure upon interaction with their binding partners [31]. Conformational flexibility also facilitates post-translational modifications as well as a fast turnover [32], both relevant mechanisms for efficient regulation. According to our results, the N-terminal region of CHOP (residues 1–99) can be divided into two modules of similar size. The first half, comprising residues 1–48, presents an essentially unstructured character, as it can be rapidly digested by different proteases. Nevertheless, we identified a segment comprising residues 15–22 (-SWELEAWY-) that exhibits propensity to form an α-helix. This segment is involved in the interaction with transcriptional regulatory proteins, such as the coactivator p300 and the repressor TRB3 [5, 6]. These authors also showed that Tyr22 plays an essential role in the ubiquitin-mediated degradation of CHOP. In addition, this α-helical propensity segment is flanked by two targets of phosphorylation by casein kinase II (Fig 2), which was shown to inactivate the transcriptional activity of CHOP [7]. Phosphorylation of serine residues within these target sequences may reduce the α-helical propensity, thus hampering the interaction of CHOP with proteins involved in transcriptional activation. Because this α-helical propensity segment is conserved in other C/EBPs (Fig 2), our findings may be extended to other members of the family. The second half of the CHOP N-terminal region, comprising residues 49–99, is less prone to proteolytic attack, suggesting that it may harbour a certain degree of higher-order structure. Accordingly, we were able to find a segment encompassing residues 57–62 (-PASLAWL-) that exhibits α-helical character. Moreover, NMR relaxation experiments confirmed that residues 55–65 present reduced flexibility with respect to neighboring CHOP residues. While this protein segment is not conserved within the C/EBP family, the detected α-helical propensity indicates that it may also play a role in CHOP function.

The two segments with α-helical propensity in the acidic N-terminal region of CHOP present a high hydrophobic content, with tryptophan appearing as central. Moreover, the residue distribution in both segments follows a pattern that represents a signature for activation domains (AD), with hydrophobic amino acids at positions i, i+3, i+4 [33]. Therefore, we propose these segments in CHOP, -SWELEAWY- and -PASLAWL-, to be referred to as AD1 and AD2, respectively. Despite the high number of residues with negative charge present in acidic ADs, their interactions with transcriptional regulators are often mediated by hydrophobic protein-protein contacts. For example, yeast transcription factor Gcn4 contains an AD that forms a short α-helix with high hydrophobic content when it interacts with the coactivator Gal11/Med15 [33]. Our results show that ADs harbor a certain α-helical character even in the absence of their binding partners, which is likely important for initial protein recognition. In addition, both ADs in CHOP are located within regions predicted to undergo disorder-to-order transitions upon interaction with other proteins (S1 Fig), a well-described phenomenon in IDPs [34]. In agreement, it has been proposed that, upon binding, coactivators induce the formation of an amphipathic α-helix in ADs [35, 36]. Based on these findings, we propose that the two α-helical segments in the N-terminal region of CHOP represent hotspots for initial recognition of their binding partners, followed by complete folding of the segments and adjacent residues to complete the disorder-to-order transition.

Apart from the two α-helical segments present in the CHOP N-terminal part, a third segment with α-helical propensity is located in the basic region of the protein (residues 99–120), involved in DNA-binding. As the DNA-binding domain is conserved among the other members of the C/EBP family, they have been proposed to adopt an α-helical fold similar to that observed for C/EBPα and C/EBPβ in the presence of DNA [10, 11]. Whereas our studies have been performed in the absence of DNA and the leucine zipper region, which are both critical for the basic region of bZIP factors to adopt an α-helical fold [12], we were able to detect a segment (residues 99–104) with α-helical character at the N-terminus of the basic region of CHOP. Within this region, computational algorithms also detect a segment predicted to fold upon interaction with its binding partner, in this case the DNA target sequence of CHOP (S1 Fig). Our results, thus, indicate that intrinsic α-helical propensity may also play a role in DNA recognition.

In summary, our data confirm that segments with secondary structure propensity, α-helical in the case of CHOP, exist within IDPs. The presence of a few structure-prone hotspots separated by flexible stretches represents an advantage to guide interactions, as previously suggested on a theoretical basis [20]. Such segments, likely representing nucleation points for binding, allow IDPs to reach larger binding distances than in the case of proteins with restricted conformational freedom, through what has been called a fly-casting mechanism [37]. The conclusions derived from our studies shed new light into the initial stages of macromolecular recognition by IDPs.

## Supporting information

S1 Fig Predicted molecular recognition features in CHOP_1-120_. The results of MoRFpred [29] and ANCHOR [30] are shown in blue and green, respectively. Threshold values of 0.4 and 0.8 for MoRFpred and ANCHOR (dotted lines) result in similar prediction of protein-protein interaction regions that likely undergo disorder-to-order transitions upon binding. A schematic representation of the segment with α-helical propensity is shown above. A vertical dotted line indicates the boundary between the N-terminal region and the basic DNA-binding domain.
(TIF)

S1 Table Experimental chemical shifts of CHOP_1-120_. (DOCX)
