# Supplementary figures and images for: Hidden α-helical propensity segments within disordered regions of the transcriptional activator CHOP
Source: PLoS One. 2017 Dec 6;12(12):e0189171. doi: 10.1371/journal.pone.0189171 (PMC5718554; doi:10.1371/journal.pone.0189171)

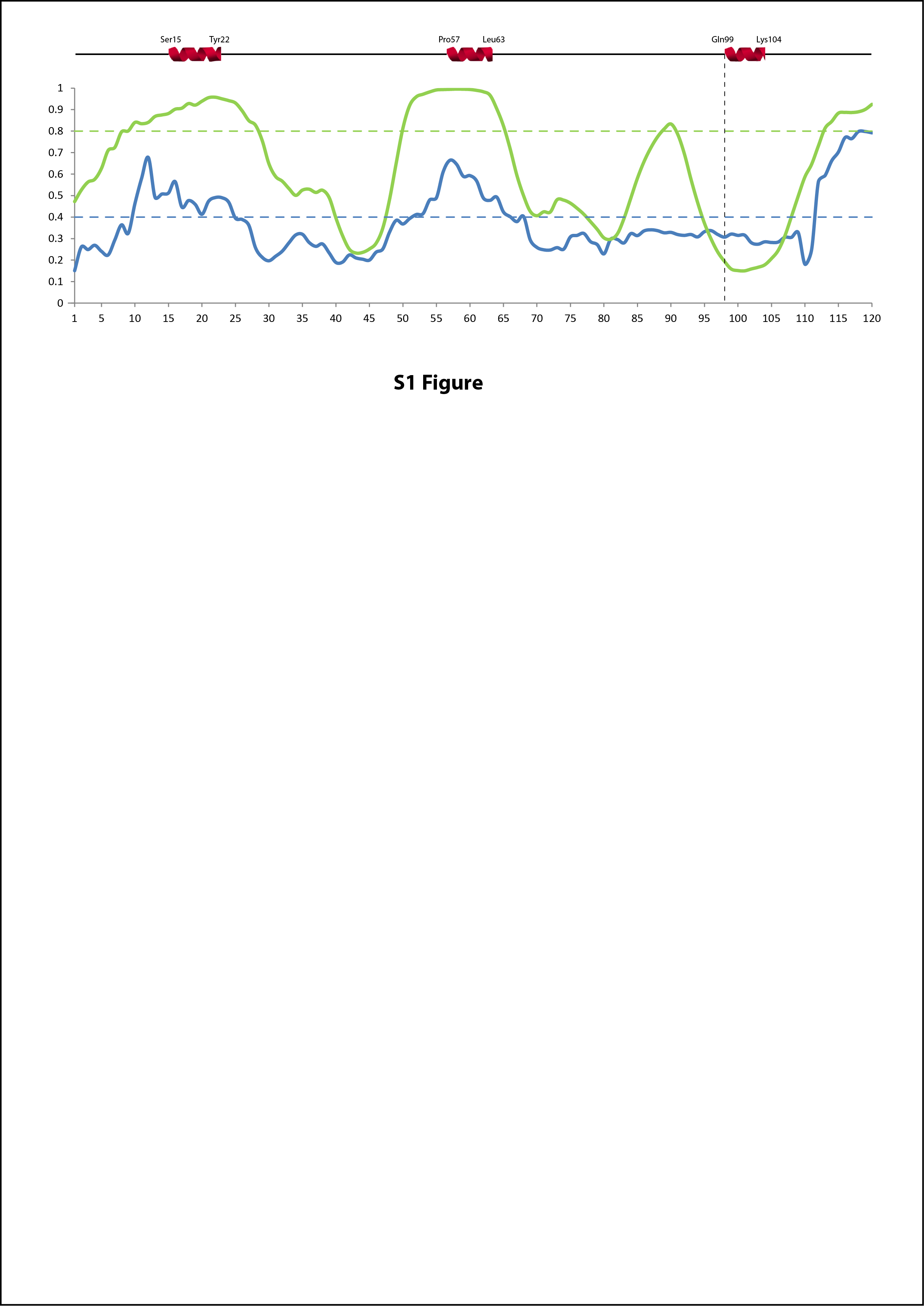

Supplement: S1 Fig — The results of MoRFpred [29] and ANCHOR [30] are shown in blue and green, respectively. Threshold values of 0.4 and 0.8 for MoRFpred and ANCHOR (dotted lines) result in similar prediction of protein-protein interaction regions that likely undergo disorder-to-order transitions upon binding. A schematic representation of the segment with α-helical propensity is shown above. A vertical dotted line indicates the boundary between the N-terminal region and the basic DNA-binding domain. (TIF) [file pone.0189171.s001.tif]
