# Supplementary material for: Hidden α-helical propensity segments within disordered regions of the transcriptional activator CHOP
Source: PLoS One. 2017 Dec 6;12(12):e0189171. doi: 10.1371/journal.pone.0189171 (PMC5718554; doi:10.1371/journal.pone.0189171)
Supplement: S1 Table — (DOCX) [file pone.0189171.s002.docx]

**S1 Table. Experimental chemical shifts of CHOP_1-120_.**

| Residue No. | Aminoacid | δ (ppm) | | | |
| --- | --- | --- | --- | --- | --- |
|  |  | H | N | CA | CB |
| -2^(a)^ | Pro |  | 133.66 | 62.81 | 31.83 |
| -1^(a)^ | Ser |  | 116.41 | 58.36 | 63.72 |
| 1 | Met |  | 122.56 | 55.01 | 32.74 |
| 2 | Ala | 8.27 | 125.23 | 52.3 | 18.99 |
| 3 | Ala | 8.27 | 123.44 | 52.44 | 18.95 |
| 4 | Glu | 8.36 | 119.2 | 56.68 | 29.93 |
| 5 | Ser | 8.19 | 116.31 | 57.87 | 63.72 |
| 6 | Leu | 8.12 | 125.01 | 52.94 | 41.67 |
| 7 | Pro |  | 135.42 | 63.06 | 31.37 |
| 8 | Phe | 8.04 | 119.65 | 57.71 | 39.17 |
| 9 | Ser | 7.96 | 116.75 |  |  |
| 10 | Phe | 8.11 | 122.25 | 58.1 | 39.31 |
| 11 | Gly | 8.26 | 109.71 | 45.1 |  |
| 12 | Thr | 7.94 | 113.47 | 61.58 |  |
| 13 | Leu | 8.08 | 124.21 | 54.97 | 42.31 |
| 14 | Ser | 8.42 | 117.03 | 58.06 | 63.72 |
| 15 | Ser | 8.34 | 117.24 | 59.24 | 63.23 |
| 16 | Trp | 7.82 | 121.59 | 57.85 | 28.6 |
| 17 | Glu | 7.79 | 121.39 | 56.71 | 29.92 |
| 18 | Leu | 7.79 | 121.14 | 55.51 | 42.09 |
| 19 | Glu | 8.04 | 119.86 | 57.21 | 29.71 |
| 20 | Ala | 7.98 | 123.04 | 52.82 | 18.62 |
| 22 | Tyr | 7.63 | 120.72 | 58.16 | 38.63 |
| 23 | Glu | 7.94 | 121.41 | 56.88 | 30.22 |
| 25 | Leu | 7.99 | 122.29 | 55.27 | 41.91 |
| 26 | Gln | 8.18 | 120.09 | 55.99 | 29.07 |
| 29 | Leu | 8.32 | 125.86 | 54.82 | 42.13 |
| 30 | Ser | 8.31 | 116.82 | 58.11 | 63.75 |
| 31 | Ser | 8.34 | 117.79 |  |  |
| 32 | Asp | 8.32 | 122.36 | 54.34 | 41.21 |
| 34 | Asn | 8.45 | 118.85 | 53.3 | 38.87 |
| 35 | Gly | 8.31 | 109.11 | 45.43 |  |
| 36 | Gly | 8.29 | 108.7 | 45.11 |  |
| 37 | Thr | 7.97 | 114.08 | 61.6 | 69.65 |
| 38 | Tyr | 8.27 | 123.79 | 57.65 | 38.83 |
| 39 | Val | 7.9 | 124.46 | 61.21 | 32.75 |
| 41 | Pro |  | 139.23 | 61.38 | 30.59 |
| 42 | Pro |  | 135.87 | 62.98 | 31.8 |
| 43 | Gly | 8.48 | 109.17 | 44.96 |  |
| 44 | Asn | 8.31 | 118.7 | 52.94 | 38.86 |
| 45 | Glu | 8.63 | 121.39 | 57.16 | 29.56 |
| 48 | Glu | 8.44 | 121.93 | 56.8 | 30.07 |
| 49 | Ser | 8.29 | 116.41 | 58.57 | 63.25 |
| 50 | Lys | 8.15 | 123.21 | 56.1 | 32.85 |
| 51 | Ile | 7.89 | 120.84 | 60.84 | 38.38 |
| 52 | Phe | 8.28 | 124.08 | 57.32 | 39.52 |
| 53 | Thr | 8.07 | 115.99 | 61.39 | 69.84 |
| 54 | Thr | 8.1 | 116.66 |  |  |
| 55 | Leu | 8.18 | 124.89 | 54.67 | 42.28 |
| 56 | Asp | 8.34 | 123.2 | 51.53 | 41.2 |
| 57 | Pro |  | 138.38 | 63.94 | 31.8 |
| 58 | Ala | 8.31 | 121.64 | 52.94 | 18.37 |
| 59 | Ser | 7.94 | 113.84 | 58.77 | 63.56 |
| 60 | Leu | 7.82 | 122.91 | 55.12 | 41.66 |
| 61 | Ala | 7.89 | 123.17 | 52.9 | 18.69 |
| 62 | Trp | 7.75 | 118.41 | 56.7 | 28.99 |
| 63 | Leu | 7.79 | 122.88 | 55.25 | 42.13 |
| 64 | Thr | 7.95 | 114.19 | 61.4 | 69.84 |
| 65 | Glu | 8.31 | 122.91 | 56.22 | 30.13 |
| 67 | Glu | 8.44 | 123.83 | 53.94 | 29.67 |
| 68 | Pro |  | 137.12 | 62.6 | 31.8 |
| 69 | Glu | 8.44 | 122.41 | 54.09 | 29.29 |
| 70 | Pro |  | 137.71 | 62.8 | 31.81 |
| 71 | Ala | 8.38 | 124.44 | 52.01 | 19.14 |
| 72 | Glu | 8.35 | 120.07 | 56.01 | 30.13 |
| 73 | Val | 8.25 | 121.8 | 62.29 | 32.44 |
| 74 | Thr | 8.25 | 117.87 | 61.52 | 69.59 |
| 75 | Ser | 8.33 | 117.91 | 57.64 |  |
| 76 | Thr | 8.22 | 115.51 | 61.59 | 69.38 |
| 77 | Ser | 8.38 | 118.16 |  |  |
| 79 | Ser | 8.35 | 118.5 | 55.98 | 63.26 |
| 80 | Pro |  | 137.94 | 62.95 | 31.81 |
| 81 | His |  | 119.33 | 55.43 | 30.27 |
| 82 | Ser |  | 118.8 |  |  |
| 83 | Pro |  | 137.82 |  | 31.86 |
| 84 | Asp | 8.32 | 119.88 | 53.88 | 40.86 |
| 85 | Ser | 8.31 | 116.86 | 58.57 | 63.72 |
| 86 | Ser | 8.42 | 118 |  |  |
| 87 | Gln | 8.28 | 121.64 | 55.8 | 29 |
| 88 | Ser | 8.26 | 116.65 | 58.54 | 63.72 |
| 89 | Ser | 8.34 | 117.93 |  | 63.75 |
| 90 | Leu | 8.15 | 123.82 | 55.03 | 42.15 |
| 91 | Ala | 8.21 | 124.62 | 52.47 | 18.7 |
| 92 | Gln | 8.28 | 119.68 | 55.84 | 29.33 |
| 98 | Asp | 8.43 | 121.44 | 54.27 | 41.15 |
| 99 | Gln | 8.51 | 120.57 | 56.69 | 28.06 |
| 100 | Gly | 8.5 | 108.44 | 45.58 |  |
| 101 | Arg | 7.99 | 120.07 | 56.71 | 30.37 |
| 102 | Thr | 8.14 | 114.06 | 62.33 | 69.3 |
| 103 | Arg | 8.28 | 123.18 | 56.2 | 30.36 |
| 104 | Lys | 8.29 | 122.08 | 56.4 | 32.69 |
| 106 | Lys | 8.4 | 122.86 | 55.87 | 32.74 |
| 108 | Ser |  | 117.31 | 58.4 | 63.74 |
| 109 | Gly |  | 110.58 | 44.95 |  |
| 110 | His |  | 119.24 | 55.76 | 30.87 |
| 111 | Ser |  | 118.86 | 56.16 | 63.27 |
| 112 | Pro |  | 137.82 | 62.95 | 31.81 |
| 113 | Ala | 8.31 | 123.88 | 52.47 | 18.97 |
| 115 | Ala | 8.28 | 125.05 | 52.36 | 19.06 |
| 116 | Gly | 8.33 | 108.2 | 44.96 |  |
| 117 | Lys | 8.17 | 120.76 | 55.95 | 32.74 |
| 118 | Gln |  | 121.71 | 55.81 | 29.49 |
| 119 | Arg | 8.38 | 123.05 | 55.98 | 30.6 |
| 120 | Met | 8.02 | 127.2 | 56.69 | 33.69 |

1. Derived from the cloning and 3C protease cleavage (see Materials and Methods for details)
